# Enabling patient-reported outcome measures in clinical trials, exemplified by cardiovascular trials

**DOI:** 10.1186/s12955-021-01800-1

**Published:** 2021-06-13

**Authors:** Theresa M. Coles, Adrian F. Hernandez, Bryce B. Reeve, Karon Cook, Michael C. Edwards, Marc Boutin, Elizabeth Bush, Arnold Degboe, Lothar Roessig, Amy Rudolph, Pauline McNulty, Nikunj Patel, Trish Kay-Mugford, Margaret Vernon, Michael Woloschak, Gustavo Buchele, John A. Spertus, Matthew T. Roe, Denise Bury, Kevin Weinfurt

**Affiliations:** 1 Duke University School of Medicine, Durham, NC USA; 2 Northwestern University Feinberg School of Medicine, Chicago, IL USA; 3 Arizona State University, Tempe, AZ USA; 4 Vector Psychometric Group, Chapel Hill, NC USA; 5 National Health Council, Washington, DC USA; 6 Eli Lilly and Company, Indianapolis, IN USA; 7 AstraZeneca, Boston, MA USA; 8 Bayer AG, Wuppertal, Germany; 9 Novartis, Bridgewater, NJ USA; 10 Janssen, Raritan, NJ USA; 11 Evidera, Bethesda, MD USA; 12 Sanofi, Bridgewater, NJ USA; 13 Ionis Pharmaceuticals Inc., Carlsbad, CA USA; 14 Saint Luke’s Mid America Heart Institute, Kansas City, MO USA; 15 University of Missouri-Kansas City, Kansas City, MO USA; 16 Sanofi, Cambridge, MA USA

**Keywords:** Patient-reported outcome measures, Clinical trials, Think Tank meeting results

## Abstract

**Objectives:**

There has been limited success in achieving integration of patient-reported outcomes (PROs) in clinical trials. We describe how stakeholders envision a solution to this challenge.

**Methods:**

Stakeholders from academia, industry, non-profits, insurers, clinicians, and the Food and Drug Administration convened at a Think Tank meeting funded by the Duke Clinical Research Institute to discuss the challenges of incorporating PROs into clinical trials and how to address those challenges. Using examples from cardiovascular trials, this article describes a potential path forward with a focus on applications in the United States.

**Results:**

Think Tank members identified one key challenge: a common understanding of the level of evidence that is necessary to support patient-reported outcome measures (PROMs) in trials. Think Tank participants discussed the possibility of creating general evidentiary standards depending upon contextual factors, but such guidelines could not be feasibly developed because many contextual factors are at play. The attendees posited that a more informative approach to PROM evidentiary standards would be to develop *validity arguments* akin to courtroom briefs, which would emphasize a compelling rationale (interpretation/use argument) to support a PROM within a specific context. Participants envisioned a future in which validity arguments would be publicly available via a repository, which would be indexed by contextual factors, clinical populations, and types of claims.

**Conclusions:**

A publicly available repository would help stakeholders better understand what a community believes constitutes compelling support for a specific PROM in a trial. Our proposed strategy is expected to facilitate the incorporation of PROMs into cardiovascular clinical trials and trials in general.

## Introduction

During a Think Tank meeting funded by the Duke Clinical Research Institute (Durham, NC) and held in July of 2018 in Washington, DC, stakeholders from academia, industry, non-profits, insurers, clinicians, and the U.S. Food and Drug Administration (FDA) considered challenges and opportunities for incorporating patient-reported outcomes (PROs) into clinical trials. For consistency throughout this article, we use cardiology-focused examples, although examples from many health conditions were discussed at the meeting. This paper describes the primary conclusions and recommendations of the authors from the multi-stakeholder scientific meeting as well as the authors’ reflections on those discussions.

The Think Tank meeting took place over 2 days and included 47 individuals who were predominantly from the United States. The meeting format consisted of alternating sessions of brief presentations describing attendees’ positions and perspectives and a discussion moderated by a senior health outcomes researcher and a clinician. Additionally, the meeting included a session in which participants responded to and discussed specific case examples. Detailed notes were recorded during the meeting describing the presentations and discussion. Presenters shared their slide sets for reference during the manuscript development. This paper summarizes the discussion and conclusions from the Think Tank meeting.

## Discussion/observations

### Benefit of incorporating PROs into clinical trials

PROs are a type of clinical outcome assessment and describe health status, such as symptoms and functional limitations, from the patient perspective. PROs are an important complement to traditional clinical outcomes, such as mortality and hospitalization, and a primary goal in optimizing the treatment of patients in clinical care. Patient-reported outcomes measures (PROMs) are patient questionnaires used to measure PROs that include patient symptoms, side effects of treatment, and the impact of symptoms and treatment on functioning and quality of life as directly elicited from patients. PROMs are the means by which PROs are tangibly evaluated and recorded. PROMs are particularly important to include in cardiovascular trials because patient experiences vary, regardless of their cardiac severity, as measured by clinical exams, labs, or imaging, and because patient experiences cannot be clearly captured via surrogate, pathophysiologic measurements. For example, two patients with heart failure may have similar hemodynamics, biomarkers, or left ventricular dimensions but present with different symptom burdens and functional limitations [1–3]. In addition, the goal of some cardiovascular therapies is not necessarily survival but to improve symptoms, function, and quality of life. Long-acting nitrates are used to reduce symptoms of angina, and ranolazine is used for relief of angina in patients with coronary artery disease. These medications have a more substantial impact on symptoms than prognosis [4, 5]. Furthermore, including PROs in clinical trials allows the often burdensome effect of a therapy to be measured and captured, thereby providing real-world patient input into the safety and efficacy of an investigational drug or device.

When included in clinical trials, PROs can provide important evidence for decision-making for a variety of stakeholders within and beyond the clinical trial space, including patients, clinicians, and health technology assessment/value assessors. For example, sponsors (a person or entity, such as a pharmaceutical company, who takes responsibility for and initiates a clinical investigation) can request a label claim [6] based on PROs to describe patient-perceived treatment benefit or tolerability; such notifications can inform prescribing and payment decisions, thereby influencing patient treatment choices. In this case, claims based on PROs require analyses and evidence (e.g., studies powered for confirmatory hypothesis testing are multiplicity controlled, alpha preserving, and hierarchically tested) in addition to evidence that the PROM is fit-for-purpose. Even if they are not seeking a PRO-based labeling claim, sponsors can choose to collect PRO data that can inform the benefit-risk decision for drug or device approval. Additionally, payers use PRO data to inform support for therapies, which affects patient and clinician treatment decisions.

Finally, PROs can provide an important dimension to inclusion/exclusion criteria in clinical trials. The primary goal of clinical trials is to define a homogenous population in which the efficacy of a new therapy can be tested. In the context of heart failure, this definition almost always involves the incorporation of New York Heart Association (NYHA) classifications. Nevertheless, the NYHA is assigned by clinicians (not patients), and inter-physician variability in assigning NYHA classes is well documented [7, 8]. The variation in clinician-defined NYHA classes versus the reliability of PRO scores [9, 10] highlights how using PROs in the inclusion/exclusion criteria of a trial might be a more reliable way of creating study populations and could facilitate more consistent application of study findings to future patients (i.e., those with PRO scores in the range of enrolled patients).

### Scientific and regulatory guidance

Through publication and initiatives, the FDA and various professional associations have encouraged the incorporation of PROMs into clinical trials for more than 10 years. One of the most influential publications is the FDA PRO guidance document (released in 2009) [11], which emphasizes the importance of using PROMs in clinical trials. Sponsors use the framework provided by the FDA PRO guidance publication to design and conduct studies that will support the inclusion of a PROM in a given trial. Specific to cardiology, professional associations, such as the American Heart Association and European Society of Cardiology, developed scientific statements calling for PROs to be included in cardiac research to complement clinical measures [2, 12]. Over the past 10 years, the International Society for Pharmacoeconomics Outcomes Research published multiple documents describing recommendations for appropriate evidence needed to support PRO-based claims [13–16] to facilitate the use of PROs in clinical trials. Internationally, outside of the United States [11] and Europe [17], some countries face hurdles justifying approval of new drugs or devices using PROs.

In addition to publications calling for the inclusion of PROs in clinical trials, the FDA has supported multiple initiatives to facilitate PROs in clinical trials, including those specified in the Prescription Drug User Fee Act (PDUFA) VI [18] and the 21st Century Cures Act. Through the year 2022, PDUFA VI performance goals have been published with priorities to collect comprehensive patient community input on disease burden and current therapy, collect impacts most important to patients, and incorporate measures evaluating such impacts in clinical trials. As part of the 21st Century Cures Act, the FDA is developing new guidance documents addressing methodological approaches to facilitate PROM incorporation into clinical trials [19]. Additionally, the FDA has published the Clinical Outcomes Assessment Compendium [20], which provides transparency on the use of PROMs (and other clinical outcomes assessments) in clinical trials for various conditions and label claims. The Center for Devices and Radiological Health published PRO case studies [21] to serve as examples and clarify potential PRO applications in clinical trials. From 2018 to 2019, the FDA solicited and awarded proposals to support the development of publicly available core sets of clinical outcomes assessments (including PROMs) and their related endpoints for specific disease indications (RFA-FD-19-006), thereby emphasizing the FDA’s desire to expedite PROM inclusion in clinical trials. Through the medical device development tool qualification process, the FDA approved the Kansas City Cardiomyopathy Questionnaire for use in studies evaluating therapies for symptomatic heart failure patients [22]. Additionally specific to cardiology, a recent update by the FDA highlights that improving symptoms and function can provide a foundation for the approval of new heart failure therapies [23].

### Challenges to incorporating PROs in trials

Despite enthusiastic support from stakeholders and organizations to integrate PROs into clinical trials, efforts to incorporate PROs into trials have been challenging for several reasons. First, there have been challenges in looking beyond the historic emphasis on “hard” outcomes, such as mortality and hospitalizations, and surrogate markers of disease progression, particularly in cardiovascular trials. Reliance on such outcomes has been motivated by the beliefs that they are more objective and unambiguous, even though they may miss important aspects of morbidity. Second, PROs present particular budget and time challenges related to generating sufficient evidence for a PRO, and unique process barriers, such as protocol implementation and site training. Third, clinical trial results reporting often does not provide PRO interpretation guidelines, which typically supply a context for PRO results. Without interpretation guidelines for PRO scores published in clinical trial manuscripts, we proliferate a sense of mystery around PROs that does not exist for other outcomes. Fourth, PROMs developed prior to FDA guidance may not have development process documentation that meets field standards; therefore, to provide psychometric evidence, further studies are required. Similarly, not all PROM development and psychometric evaluation studies conducted or supported by sponsors are published, leading to parallel development of PROMs, making it more time-consuming and costly to include PROs in trials. Fifth, even with the PRO guidance, stakeholders are still uncertain about what and how much evidence is necessary to support using PROMs for efficacy evaluation in specific clinical trials. Over time, the 2009 FDA guidance was often interpreted as a prescriptive list of expectations, procedures, and methods that must be applied (e.g., item-tracking matrices, saturation grids) and properties that must be evaluated in every case. However, a rigid interpretation of this publication does not reflect the nuances of PROM validity evidence (e.g., seriousness of the condition, prevalence of the condition, level of unmet need, endpoint positioning, availability of PROMs, age of participants, comorbidity dependencies of PROMs). As a result, some sponsors have concluded that the FDA’s feedback on psychometric evidence is inconsistent. Finally, various review divisions within the FDA may have approached inclusion and review of PROMs differently over time, thereby making it challenging for sponsors to decipher priorities for PROM evidence generation.

### Context matters

The primary challenge addressed during the Think Tank meeting was the importance of context in deciding which (and how much) evidence should be provided to support the use of a PROM in a clinical trial. For example, consider rare cardiovascular diseases: The available sample size for qualitative and psychometric studies would be smaller for a rare cardiovascular disease trial than a trial involving a more prevalent cardiovascular condition. Low sample size reduces the ability to obtain saturation in qualitative studies or to have confidence in the psychometric results. Additionally, there are difficulties involved with conducting multiple trials in small populations; as a result, the available level of evidence to support a PROM may be less robust than for more prevalent conditions. Other contextual factors also influence what information can/should be provided to support the use of PROMs in clinical trials. Notably, contextual factors differ from the context of use. Per the FDA-National Institutes of Health Biomarker Working Group, context of use is defined as “a statement that fully and clearly describes the way the medical product development tool is to be used and the medical product development-related purpose of the use.” Contextual factors that may influence what information can and should be provided in support of PROM include seriousness of the condition (whether life-threatening [e.g., stage D heart failure] or not serious [e.g., palpitations]), level of unmet need (availability of an already approved therapy; remaining disease burden despite available standard of care), current availability of a fit-for-purpose PROM (there was some variability among Think Tank participants in their understanding of whether suitable fit-for-purpose PROMs were available for a given condition), endpoint positioning (whether the PROM supports a primary, secondary, or exploratory endpoint), and possible comorbidity dependency of the PRO. Consequently, the evidence that is needed to support using a PROM may differ depending on the contextual factors. Meeting participants discussed the possibility of creating general recommendations for evidentiary standards depending on specific contextual factors, but it became clear that such guidelines could not be feasibly developed due to the vast number of contextual factors at play.

### Validity arguments instead of validity checklists

Many Think Tank participants noted the similarities between the challenges found in providing PROM evidence and those faced in a court of law; more specifically, participants noted parallels between PRO evidence and case law. Building on an analogy from Bruno Zumbo [24], courtroom decisions for a defendant or plaintiff cannot be effectively managed using an evidence checklist; rather, context is critical and must be considered when weighing evidentiary findings. Previous courtroom cases provide insight for judges and juries, acting as a guide when evaluating evidential weight in future cases. Attorneys often submit briefs, which are narratives that justify a given stance or a reaction to evidence or questions. The courtroom metaphor led many Think Tank members to reevaluate current approaches to submitting and assessing evidence for the use of PROMs in clinical trials. Think Tank members posited that a more informative and nuanced approach to address challenges (outlined in previous sections of this paper) would be to develop validity arguments*.* Consistent with modern validity theory [25–31], a validity argument refers to a compelling rationale for a proposed interpretation and use of a PROM within a given context of use. Akin to making an argument in a court of law, the type and amount of supportive evidence needed would be determined by the specific argument being made. The decision that a PROM is suitable for a proposed interpretation and use would be made by evaluating the logic, coherence, and quality of evidentiary support for the validity argument. Building on the FDA’s efforts to facilitate transparency with the Clinical Outcome Assessment Compendium [20] and Center for Devices and Radiological Health PRO case studies [21], validity arguments could be made publicly available. Similar to legal archives, the accumulation of successful validity arguments (i.e., a validity argument repository) would provide insight on the level of evidence needed for particular contexts. To assist users, such a repository could be indexed by contextual factors, clinical populations, and types of claims. A publicly available repository would help stakeholders better understand what the community believes constitutes a compelling validity argument. Users of such a repository (e.g., patient organizations, academia, industry, payers, and regulatory agencies) would be able to determine precedents, as well as the circumstances in which those precedents might be overturned. Movement toward the use of validity arguments would promote explicit discussion within the community about what constitutes compelling justifications—a discussion that is, ideally, informed by the growing repository of publicly available examples. One of the authors of this commentary has taken the idea of validity arguments a step further by outlining assumptions for the use and interpretation of PROM scores in research settings [32]. Further experience and discussion are needed to explore the best approaches to efficiently and clearly convey validity arguments and their supporting evidence, and apply them in complex clinical trial planning and design.

When considering the courtroom analogy, it is important to note that each court case involves a confluence of different contextual factors, so that arguments for guilt or innocence may vary on a case-by-case basis. Likewise, a PROM may be acceptable for one cardiovascular condition given certain contextual factors, but not acceptable for the same cardiovascular condition given different contextual factors. Additionally, similar to legal cases, PROMs will not need to be retried if evidence already supports the use of a particular PROM in a similar context. Nonetheless, stakeholders cannot become complacent with PROM development; as psychometric methods, science, and technology advance, there will be ample opportunity to develop new PROMs and improve the old.

As validity arguments are incorporated, relevant conditions can routinely and efficiently integrate PROMs for regulatory decision-making. For example, as PROMs are used more frequently for common conditions such as heart failure, a precedent may evolve regarding what is expected for including PROMs in clinical trials and labeling in this context. Similarly, for rare conditions, such as hypertrophic cardiomyopathy, the accumulation of publicly available documentation on context and regulatory acceptance of PROM evidence will provide clarity over time. The validity argument approach for the use of PROMs might also be extended to decisions surrounding other sources of patient outcomes, such as data derived from wearables or mobile health (mHealth)-derived endpoints.

## What questions remain

More work is needed to address important practical questions that remain related to the Think Tank’s overall proposal. For example, how can validity arguments best be communicated to regulators? Can a template of a validity argument for a PRO measure be developed? [32] How would a repository of validity arguments be managed and maintained, and what rules would be needed to ensure transparency without compromising a sponsor’s competitive information? Would the repository include validity arguments that regulators judged to be unacceptable as well as those considered acceptable?

Any changes to the current review process would require more up-front effort from both regulators and sponsors. However, would use of validity arguments ultimately result in more efficient preparation and review of submissions to regulatory agencies?

The validity argument approach suggested by the Think Tank participants is largely based on the perspectives of individuals from the United States, and a remaining question is how this approach could be integrated in other countries with different levels of emphasis on PROs. Future work should explore how the validity argument approach would be best integrated for regulatory agencies outside of the FDA.

## Conclusions

Think Tank participants explored the challenges and complexities associated with the evidence required to support the inclusion of PROMs in clinical trials and examined various approaches that would best enable PROMs to be incorporated into clinical trials. With promotion and application of modern validity theory, clinical trials could move toward adopting context-specific validity arguments for PROMs. Collecting and indexing the arguments as a community resource could create greater consensus on the evidence that is necessary for justifying PROM use in common health contexts. This community-inspired consensus should accelerate patient-centered medical product development and promote innovation. Moving toward a modern validity theory approach will require additional leadership from regulatory agencies, industry, and researchers, as well as the submission of narrative justifications by industry. Stakeholders will be more likely to incorporate PROMs into clinical trials if they are provided with a clear, evidence-generated foundation upon which to build.

## Data Availability

Data sharing is not applicable to this article as no datasets were generated or analyzed during the current study.

## Abbreviations

FDA: U.S. Food and Drug Administration
NYHA: New York Heart Association
PROs: Patient-reported outcomes
PROMs: Patient-reported outcome measures

## References

[1] Lee CS, Hiatt SO, Denfeld QE, Mudd JO, Chien C, Gelow JM (2015). Symptom-hemodynamic mismatch and heart failure event risk. J Cardiovasc Nurs.

[2] Rumsfeld JS, Alexander KP, Goff DC, Graham MM, Ho PM, Masoudi FA (2013). Cardiovascular health: the importance of measuring patient-reported health status: a scientific statement from the American Heart Association. Circulation.

[3] Luther SA, McCullough PA, Havranek EP, Rumsfeld JS, Jones PG, Heidenreich PA (2005). The relationship between B-type natriuretic peptide and health status in patients with heart failure. J Card Fail.

[4] Tarkin JM, Kaski JC (2018). Nicorandil and long-acting nitrates: vasodilator therapies for the management of chronic stable angina pectoris. Eur Cardiol.

[5] Scirica BM (2007). Ranolazine in patients with coronary artery disease. Expert Opin Pharmacother.

[6] U.S. Food and Drug Administration (FDA). Label claims for conventional foods and dietary supplements. U.S. FDA web site. https://www.fda.gov/food/food-labeling-nutrition/label-claims-conventional-foods-and-dietary-supplements. Updated June 19, 2018. Accessed 26 Apr 2021.

[7] Taichman DB, McGoon MD, Harhay MO, Archer-Chicko C, Sager JS, Murugappan M (2009). Wide variation in clinicians' assessment of New York Heart Association/World Health Organization functional class in patients with pulmonary arterial hypertension. Mayo Clin Proc.

[8] Raphael C, Briscoe C, Davies J, Whinnett ZI, Manisty C, Sutton R (2007). Limitations of the New York Heart Association functional classification system and self-reported walking distances in chronic heart failure. Heart.

[9] Spertus JA, Jones PG (2015). Development and validation of a short version of the Kansas City Cardiomyopathy Questionnaire. Circ Cardiovasc Qual Outcomes.

[10] Ahmad FS, Kallen MA, Schifferdecker KE, Carluzzo KL, Yount SE, Gelow JM (2019). Development and initial validation of the PROMIS^®^-Plus-HF Profile Measure. Circ Heart Fail.

[11] U.S. Department of Health and Human Services, Food and Drug Administration, Center for Drug Evaluation and Research, Center for Biologics Evaluation Research, Center for Devices and Radiological Health. Guidance document: patient-reported outcome measures, use in medical product development to support labeling claims. U.S. FDA web site. https://www.fda.gov/downloads/drugs/guidances/UCM193282.pdf. Published December 2009. Accessed 13 Nov 2019.

[12] Anker SD, Agewall S, Borggrefe M, Calvert M, Caro JJ, Cowie MR (2014). The importance of patient-reported outcomes: a call for their comprehensive integration in cardiovascular clinical trials. Eur Heart J.

[13] Rothman M, Burke L, Erickson P, Leidy NK, Patrick DL, Petrie CD (2009). Use of existing patient-reported outcome (PRO) instruments and their modification: the ISPOR Good Research Practices for Evaluating and Documenting Content Validity for the Use of Existing Instruments and Their Modification PRO Task Force Report. Value Health.

[14] Benjamin K, Vernon MK, Patrick DL, Perfetto E, Nestler-Parr S, Burke L (2017). Patient-reported outcome and observer-reported outcome assessment in rare disease clinical trials: an ISPOR COA Emerging Good Practices Task Force Report. Value Health.

[15] Patrick DL, Burke LB, Gwaltney CJ, Leidy NK, Martin ML, Molsen E (2011). Content validity—establishing and reporting the evidence in newly developed patient-reported outcomes (PRO) instruments for medical product evaluation: ISPOR PRO Good Research Practices Task Force report: part 2—assessing respondent understanding. Value Health.

[16] Patrick DL, Burke LB, Gwaltney CJ, Leidy NK, Martin ML, Molsen E (2011). Content validity—establishing and reporting the evidence in newly developed patient-reported outcomes (PRO) instruments for medical product evaluation: ISPOR PRO good research practices task force report: part 1—eliciting concepts for a new PRO instrument. Value Health.

[17] European Medicines Agency. ICH reflection paper on proposed ICH guideline work to advance patient focused drug development. https://www.ema.europa.eu/en/documents/scientific-guideline/ich-reflection-paper-proposed-ich-guideline-work-advance-patient-focused-drug-development_en.pdf. Published December 10, 2020. Accessed 26 Apr 2021.

[18] U.S. Food and Drug Administration (FDA). Prescription Drug User Fee Act (PDUFA) VI: Fiscal Years 2018 - 2022. U.S. FDA web site. https://www.fda.gov/industry/prescription-drug-user-fee-amendments/pdufa-vi-fiscal-years-2018-2022. Updated May 9, 2019. Accessed 14 Nov 2019.

[19] U.S. Food and Drug Administration (FDA). 21st Century Cures Act. U.S. FDA web site. https://www.fda.gov/regulatoryinformation/lawsenforcedbyfda/significantamendmentstothefdcact/21stcenturycuresact/default.htm. Updated March 29, 2018. Accessed 13 Nov 2019.

[20] U.S. Food and Drug Administration (FDA). Clinical outcome assessment compendium. U.S. FDA web site. https://www.fda.gov/drugs/development-resources/clinical-outcome-assessment-compendium. Updated August 21, 2019. Accessed 13 Nov 2019.

[21] U.S. Center for Devices and Radiological Health, Food and Drug Administration (FDA). Value and use of patient-reported outcomes in assessing effects of medical devices: CDRH strategic priorities 2016–2017. U.S. FDA web site. https://www.fda.gov/media/109626/download. Accessed 13 Nov 2019.

[22] U.S. Food and Drug Administration (FDA). Medical device development tool (MDDT) qualification decision summary for Kansas City Cardiomyopathy Questionnaire (KCCQ). U.S. FDA web site. https://www.fda.gov/downloads/MedicalDevices/ScienceandResearch/MedicalDeviceDevelopmentToolsMDDT/UCM581761.pdf. Accessed 13 Nov 2019.

[23] U.S. Food and Drug Administration (FDA). FDA in brief: FDA takes steps to spark development of heart failure drugs. U.S. FDA web site. https://www.fda.gov/news-events/fda-brief/fda-brief-fda-takes-steps-spark-development-heart-failure-drugs. Updated June 27, 2019. Accessed 13 Nov 2019.

[24] Zumbo BD, Rao CR, Sinharay S (2006). Validity: foundational issues and statistical methodology. Handbook of statistics.

[25] Edwards MC, Slagle A, Rubright JD, Wirth RJ (2018). Fit for purpose and modern validity theory in clinical outcomes assessment. Qual Life Res.

[26] Hawkins M, Elsworth GR, Osborne RH (2018). Application of validity theory and methodology to patient-reported outcome measures (PROMs): building an argument for validity. Qual Life Res.

[27] Cook DA, Brydges R, Ginsburg S, Hatala R (2015). A contemporary approach to validity arguments: a practical guide to Kane's framework. Med Educ.

[28] Hatala R, Cook DA, Brydges R, Hawkins R (2015). Constructing a validity argument for the Objective Structured Assessment of Technical Skills (OSATS): a systematic review of validity evidence. Adv Health Sci Educ.

[29] Hill HC, Charalambous CY, Blazar D, McGinn D, Kraft MA, Beisiegel M (2012). Validating arguments for observational instruments: attending to multiple sources of variation. Educ Assess.

[30] Reeves TD, Marbach-Ad G (2016). Contemporary test validity in theory and practice: a primer for discipline-based education researchers. CBE Life Sci Educ.

[31] Royal KD (2017). Four tenets of modern validity theory for medical education assessment and evaluation. Adv Med Educ Pract.

[32] Weinfurt KP (2021). Constructing arguments for the interpretation and use of patient-reported outcome measures in research: an application of modern validity theory. Qual Life Res.

